# Madecassoside inhibits estrogen deficiency‐induced osteoporosis by suppressing RANKL‐induced osteoclastogenesis

**DOI:** 10.1111/jcmm.13942

**Published:** 2018-10-19

**Authors:** Qingqing Wang, Lingya Yao, Ke Xu, Haiming Jin, Kai Chen, Ziyi Wang, Qian Liu, Zhen Cao, Jacob kenny, Yuhao Liu, Jennifer Tickner, Huazi Xu, Jiake Xu

**Affiliations:** ^1^ Department of Orthopaedics The Second Affiliated Hospital and Yuying Children's Hospital of Wenzhou Medical University Zhejiang China; ^2^ School of Biomedical Sciences The University of Western Australia Perth Western Australia Australia; ^3^ Research Centre for Regenerative Medicine and Guangxi Key Laboratory of Regenerative Medicine Guangxi Medical University Guangxi China; ^4^ Department of Biomedical Materials Science Third Military Medical University Chongqing China; ^5^ The Lab of Orthopaedics and Traumatology of Lingnan Medical Research Center Guangzhou University of Chinese Medicine Guangzhou China

**Keywords:** MAPK, NFATc1, NF‐κB, Osteoclast, OVX

## Abstract

Osteoporosis is the most common osteolytic disease characterized by excessive osteoclast formation and resultant bone loss, which afflicts millions of patients around the world. Madecassoside (MA), isolated from *Centella asiatica*, was reported to have anti‐inflammatory and antioxidant activities, but its role in osteoporosis treatment has not yet been confirmed. In our study, MA was found to have an inhibitory effect on the RANKL‐induced formation and function of OCs in a dose‐dependent manner without cytotoxicity. These effects were attributed to its ability to suppress the activity of two transcription factors (NFATc1 and c‐Fos) indispensable for osteoclast formation, followed by inhibition of the expression of bone resorption‐related genes and proteins (Acp5/TRAcP, CTSK, ATP6V0D2/V‐ATPase‐d2, and integrin β3). Furthermore, we examined the underlying mechanisms and found that MA represses osteoclastogenesis by blocking Ca^2+^ oscillations and the NF‐κB and MAPK pathways. In addition, the therapeutic effect of MA on preventing bone loss in vivo was further confirmed in an ovariectomized mouse model. Therefore, considering its ability to inhibit RANKL‐mediated osteoclastogenesis and the underlying mechanisms, MA might be a potential candidate for treating osteolytic bone diseases.

## 1 INTRODUCTION

Bone undergoes continuous remodelling to maintain its homeostasis by regulating the balance between osteoblasts and osteoclasts (OCs).^1^ The disequilibration of this homeostasis, in favour of sthenic bone resorption caused by excess number or activity of mature OCs, can result in a myriad of skeletal lytic diseases, such as osteopetrosis,^2^ Paget's disease^3^ and osteoporosis.^4^ Amongst these diseases, the high morbidity of osteoporosis makes it a serious public health concern, as approximately 40% of women older than 50 will suffer from osteoporosis, which eventually leads to increased risk of pathological fractures.^5^ Current drugs for clinical use in treating osteoporosis, such as bisphosphonates, calcitonin, and estrogen, help maintain bone protein and mineral content and reduce fractures.^6^ However, serious side effects such as breast and endometrial cancer and hypercalcemia limit the use of these pharmacological drugs.^7^, ^8^ Natural compounds with fewer side effects are more suitable than synthetic drugs for treating chronic diseases that require long‐term treatment.^9^, ^10^, ^11^ Therefore, exploration of natural compounds that have therapeutic effects targeting the differentiation and function of OCs is urgently needed.

OCs are multinucleated cells that originate from bone marrow monocytes (BMMs).^12^ The process of this differentiation is mainly regulated by two osteoblast‐derived factors: receptor activator of nuclear factor κ‐B ligand (RANKL) and macrophage colony‐stimulating factor (M‐CSF).^13^ M‐CSF functions both to maintain the proliferation of osteoclast precursor (OCP) cells and to stimulate RANK expression.^14^ RANKL plays a major role in the differentiation of OCP cells. Once RANKL combines with RANK on the membrane of a preosteoclast, adaptor molecules, such as TNF receptor‐associated factor, will be recruited to stimulate the nuclear factor κ‐B (NF‐κB), mitogen‐activated protein kinase (MAPK) and calcium signaling pathways for OC differentiation and formation.^15^, ^16^, ^17^ Then, the two main transcription factors in osteoclast differentiation, activator protein 1 (AP‐1) and nuclear factor of activated T cells, cytoplasmic 1 (NFATc1), are triggered to promote preosteoclast differentiation and improve the expression of OC function‐related genes and proteins, including tartrate‐resistant acid phosphatase (TRAcP/acp5), cathepsin K (CTSK), V‐ATPase d2 (ATP6V0D2) and integrin β3).^17^, ^18^, ^19^ Therefore, if steps in RANKL‐induced osteoclastogenesis can be suppressed, skeletal lytic diseases may have the potential to be cured.

Madecassoside (MA), a pentacyclic triterpenoid saponin isolated from *Centella asiatica,* 20 exhibits various bioactivities, including antioxidative and anti‐inflammatory.^21^ Treatment with MA can significantly reduce myocardial ischemia‐reperfusion injury and lipid peroxidation.^22^, ^23^ Furthermore, MA was also found to have inhibitory effects on the NF‐κB and ERK/p38 pathways, leading to a reduction in oxidative stress and suppression of inflammatory responses.^24^ However, the effects of MA on osteoclast differentiation and function have not yet been reported. Since the NF‐κB and ERK/p38 pathways are also instrumental in RANKL‐induced osteoclastogenesis, we studied MA‐mediated regulation of RANKL‐stimulated osteoclastogenesis from BMMs to mature OCs and its underlying mechanism.

In this study, to examine the role of MA in osteoclast differentiation and function, multimodal measurements (including the number and function of OCs, the expression of OC‐related genes and proteins, etc.) were performed. Particularly, we focused on the effects of MA on the activities of two main transcription factors (NFATc1 and c‐Fos), which control the terminal differentiation of OCs, and their underlying regulatory mechanisms, such as Ca^2+^ oscillations and the NF‐κB and MAPK signaling pathways. Furthermore, an estrogen deficiency‐induced osteoporosis mouse model was used to confirm the therapeutic effect of MA on preventing bone loss in vivo. Our data provide exciting results indicating that MA might be a potential treatment option for osteolytic bone diseases.

## 2 MATERIALS AND METHODS

### 2.1 Materials and reagents

MA (purity >98%) was obtained from the National Institutes for Food and Drug Control (Beijing, China) and dissolved in dimethyl sulfoxide (DMSO) at a stock concentration of 100 mmol L^−1^. Alpha modified minimal essential medium (α‐MEM), penicillin/streptomycin (P/S), and fetal bovine serum (FBS) were purchased from Thermo Fisher Scientific (Carlsbad, CA, USA). M‐CSF and GST‐rRANKL were produced in our laboratory according to previous reports.^25^, ^26^ Tetrazolium salt (MTS) solution, TRIzol, and rhodamine‐conjugated phalloidin were purchased from Thermo Fisher Scientific (San Jose, CA, USA). Oligo‐dT primer and SYBR Green were obtained from Imgenex (Littleton, CO, USA). Primary antibodies specific for NFATc1, integrin β3, Cathepsin K, V‐ATPase‐d2, IκB‐α, pERK1/2, ERK1/2, and β‐actin were obtained from Santa Cruz Biotechnology (San Jose, CA, USA). Antibodies specific for c‐Fos, pJNK1/2, JNK1/2, p‐p38, and p38 were purchased from Cell Signaling Technology (Beverly, MA, USA).

### 2.2 Cell culture and cytotoxicity assays

BMMs were extracted from the long bone marrow of C57BL/6 mice at 10 weeks of age using procedures approved by the Animal Ethics Committee of the University of Western Australia (RA/3/100/1244). Then, BMMs were cultured in complete α‐MEM medium containing 10% (v/v) FBS, 1% (v/v) P/S, and 50 ng/mL M‐CSF, which was changed every 2 days.

### 2.3 Drug screening and cytotoxicity assays

BMMs at passage 2 were used to screen the effectiveness of drugs at suppressing RANKL‐induced osteoclastogenesis. Cells were seeded into 96‐well plates at a concentration of 5 × 10^3^ cells per well in complete α‐MEM medium prepared with M‐CSF (50 ng/mL) and were cultured overnight to adhere. Then, the BMMs were stimulated with GST‐rRANKL (50 ng/mL). Additionally, 10 μmol L^−1^ of natural compounds was added to screen for effective drugs. The complete medium was changed every 2 days, accompanied by the addition of fresh GST‐rRANKL and compounds until OCs formed on the sixth day. The cells were fixed with 2.5% glutaraldehyde for 15 min and stained with TRAcP staining solution. TRAcP‐positive multinucleated cells that had more than three nuclei were counted as OCs and used to evaluate the effect on inhibiting formation. After that, cytotoxicity assays were performed. BMMs were seeded into 96‐well plates at a concentration of 5 × 10^3^ cells per well and left overnight until the cells were confluent. Then, the cells were treated with increasing concentrations of MA (1 μmol L^−1^, 2.5 μmol L^−1^, 5 μmol L^−1^, and 10 μmol L^−1^) for 5 days. Next, 20 μl MTS solution (Thermo Fisher Scientific) was added to each well and incubated with the BMMs for another 2 h. The absorbance at 490 nm was read by a microplate reader (Multiskan Spectrum; Thermo Labsystems, Chantilly, VA, USA).

### 2.4 Immunofluorescence staining

A total of 5 × 10^3^ BMMs per well were cultured in a 96‐well plate with M‐CSF (50 ng/mL) for 24 h. As mentioned above, the cells were then treated with GST‐rRANKL (50 ng/mL) for 6 days to form mature OCs with or without varying concentrations (5 μmol L^−1^ or 10 μmol L^−1^) of MA. After that, the cells were fixed for 10 min and blocked for 20 min using 4% paraformaldehyde and 5% bovine serum albumin, respectively. Next, the cells were probed with rhodamine‐conjugated phalloidin (Thermo Fisher Scientific) for 45 min to stain for F‐actin. After being washed with PBS and stained with DAPI, cells were visualized on a confocal fluorescence microscope (Nikon, A1 PLUS, Tokyo, Japan) at 100× magnification.

### 2.5 Hydroxyapatite resorption assay

The hydroxyapatite resorption assay was used to measure the function of the induced OCs as described previously.^27^ BMMs were first seeded into a 6‐well collagen‐coated plate (BD Biosciences, Sydney, Australia) with 1 × 10^5^ cells in each well and then were stimulated with M‐CSF (50 ng/mL) and GST‐rRANKL (100 ng/mL) every 2 days to allow OCs to form. Next, the cells were dissociated from the collagen plate, and equal numbers of cells were transferred to the wells of a hydroxyapatite‐coated plate (CLS3989, Corning, NY, USA). Mature OCs were cultured in complete medium with GST‐rRANKL (50 ng/mL) and M‐CSF (50 ng/mL) in the presence or absence of MA (5 μmol L^−1^ or 10 μmol L^−1^). After 2‐3 days, the wells were separated into two groups. One group was used to count the number of multinucleated cells in each well by TRAcP staining, as described above. The other group was used to measure the resorbed areas by bleaching for 10 min and removing the cells from the wells. The resorbed areas were captured by microscopy and analysed by ImageJ software (NIH, Bethesda, MD, USA) to count the percentage of resorbed area by the OCs.

### 2.6 Luciferase reporter assays

RAW264.7 cells (American Type Culture Collection, Manassas, VA, USA) were stably transfected with two kinds of luciferase reporter construct (p‐NF‐κB‐TA‐Luc and p‐NFAT‐TA‐Luc), which separately respond to NF‐κB and NFATc1.^28^, ^29^, ^30^ Equal numbers of 1.5 × 10^5^ transfected cells were seeded into each well of a 48‐well plate to adhere overnight and then were pretreated with varying densities of MA (1, 2.5, 5, and 10 μmol L^−1^) for 1 h. Then, the cells were stimulated with 50 ng/mL GST‐rRANKL for 6 h (for measurement of NF‐κB) or 24 h (for measurement of NFAT). Cells were then lysed for luciferase activity analysis using the Promega luciferase kit and BMG Polar Star Optima luminescence reader (BMG; Labtech, Offenburg, Germany) as previously described.^31^

### 2.7 Quantitative real‐time PCR analysis

A total of 5 × 10^3^ BMMs per well were plated in a 6‐well plate in the presence of GST‐rRANKL (50 ng/mL) and M‐CSF (50 ng/mL) with or without various densities of MA for 5 days. As described in our previous study,^32^ TRIzol (Qiagen, Hilden, Germany) was used to extract total RNA from cells. With an oligo‐dT primer, single‐stranded cDNA was reverse transcribed from 2 μg total RNA. The resulting cDNA was then used for real‐time PCR based on SYBR Green (Imgenex, Littleton, CO, USA) with the specific primers displayed in Table 1. The expression level was normalized to Hmbs expression. The fold change was determined using the Livak equation, and the ratios compared to the vehicle group were calculated.

**Table 1:** Primer sequences used in qRT‐PCR

Genes	Forward (5′→3′)	Reverse (5′→3′)	Tm (°C)
Nfatc1	GGAGAGTCCGAGAATCGAGAT	TTGCAGCTAGGAAGTACGTCT	60
C‐fos	GCGAGCAACTGAGAAGAC	TTGAAACCCGAGAACATC	60
Acp5	TGTGGCCATCTTTATGCT	GTCATTTCTTTGGGGCTT	59
V‐ATpase‐d2	AGCAGACTACCTGAGTTTGAACC	GCTGCATCTGAAGAATGCGGTG	60
Hprt	GTTGGGCTTACCTCACTGCT	TAATCACGACGCTGGGACTG	60

### 2.8 Western blot

To examine the expression of bone resorption‐related proteins or the NFATc1 signaling pathway, BMMs were seeded (1 × 10^5^ cells/well) into 6‐well plates and incubated with or without MA (10 μmol L^−1^) in the presence of GST‐rRANKL (50 ng/mL) for 5 days. Cells were then lysed, and total protein was harvested using radioimmune precipitation assay (RIPA) lysis buffer (containing 100 g/mL PMSF, 500 g/mL DNase I and phosphatase inhibitors) at the following time points: 0, 1, 3, and 5 days. For short time point signaling pathways, BMMs at 5 × 10^5^ cells/well were seeded into 6‐well plates and incubated in complete medium (50 ng/mL M‐CSF) overnight. The next day, cells were starved for 4 h and then pretreated with MA for 2 h. After that, 50 ng/mL GST‐rRANKL was added to the wells, and total protein was harvested by RIPA lysis at the following time points: 0, 10, 20, 30, and 60 min. The protein was loaded and separated by 10% sodium dodecyl sulfate‐polyacrylamide gel electrophoresis. Next, the separated proteins were transferred onto nitrocellulose membranes (Whatman, Florham Park, NJ, USA) and were blocked in 5% skim milk for 1 h. The membranes were incubated overnight at 4°C with the following primary antibodies: anti‐NFATc1 (1:1000, Cat# sc‐7294, RRID:AB_2152503), anti‐c‐Fos (1:2000, Cat# 2250, RRID:AB_2247211), anti‐integrin β3(1:1000, Cat# sc‐6617‐R, RRID:AB_2129625), anti‐CTSK (1:2000, Cat# sc‐48353, RRID:AB_2087687), anti‐V‐ATPase‐d2 (1:1000, Cat# sc‐69108, RRID:AB_10406172), anti‐IκB‐α 1 (1:1000, Cat# sc‐371, RRID:AB_2235952), anti‐pERK1/2 (1:1000, Cat# sc‐16564, RRID:AB_2140540), anti‐ERK1/2 (1:1000, Cat# sc‐514302, RRID:AB_2571739), anti‐pJNK1/2 (1:1000, Cat# 9251, RRID:AB_331659), anti‐JNK1/2 (1:2000, Cat# 9252, RRID:AB_2250373), anti‐p‐p38 (1:1000, Cat# 4511L, RRID:AB_2139679), anti‐p38 (1:1000, Cat# 9212, RRID:AB_330713), and anti‐β‐actin (1:3000, Cat# sc‐47778, RRID:AB_626632). Later, the membranes were incubated for 1 h with the corresponding horseradish peroxidase‐conjugated secondary antibodies. Finally, the membranes were treated with Enhanced Chemiluminescence reagents (Amersham, Piscataway, NJ, USA) according to the manufacturer's instructions. Images were visualized using an Image quant LAS 4000 (GE Healthcare, Sydney, Australia).

### 2.9 Ca^2+^ oscillation measurement

BMMs (3 × 10^4^) were seeded into a 48‐well plate with treatments added based on different groups. In the treatment group, cells were treated with GST‐rRANKL (50 ng/mL), M‐CSF (50 ng/mL), and MA (10 μmol L^−1^); in the positive control group, cells were treated with RANKL (50 ng/mL) and M‐CSF (50 ng/mL) but without MA; and in the control group, cells were treated only with M‐CSF (50 ng/mL). After being cultured for 24 h, the cells were washed twice with Assay buffer (HANKS buffer dissolved with 1 mmol L^−1^ probenecid and 2% FCS) and stained with Fluo‐4 staining solution (Fluo‐4 AM dissolved in 20% (w/v) Pluronic F‐127 in DMSO added to Assay buffer) in the dark at 37°C for 45 min. When the staining was finished, the cells were rinsed again with Assay buffer and incubated at room temperature in the dark for 20 min after removal of the staining solution. The intensity of fluorescence was observed under fluorescent light (at an excitation wavelength of 488 nm) by inverted fluorescence microscope (Nikon). Images were captured every 2 s for 4 min. Cells with at least two oscillations were counted as oscillating cells. The average amplitude of each oscillating cell was analysed by Nikon Basic Research Software (Nikon).

### 2.10 Mouse ovariectomy (OVX) procedures

Female C57BL/6 mice (10 weeks, n = 30) were provided by the Animal Center of the Chinese Academy of Science (Shanghai, China) and were randomly divided into three groups: a sham group (n = 10), OVX group (n = 10) and OVX+MA group (n = 10) (10 mg/kg). All the mice were kept in individual ventilated cages (IVC, five rats per cage) in a specific‐pathogen‐free (SPF) room. After a week of adjustable feeding, ovariectomy based on a previously described method 27 was performed for the OVX group and OVX+MA group, whereas a sham operation was performed for the sham group as a control. Seven days later after the surgery, intraperitoneal injections of MA (10 mg/kg) for the OVX+MA group and PBS for the sham and OVX groups were given every 2 days for a total of 6 weeks.^27^ Then, the mice were all sacrificed, and the femurs were removed for histological and micro‐CT (μCT) analysis as previously described.^27^, ^29^

### 2.11 Micro‐CT scanning

The femur samples were fixed with 4% paraformaldehyde for 24 h and analysed by a Skyscan 1176 micro‐CT instrument (Skyscan, Bruker, Belgium). Images were acquired using a 50‐kV X‐ray tube voltage, a 500‐μA current, an isotropic pixel size of 9 μmol L^−1^ (1600 × 2672‐pixel image matrix) and a 0.5‐mm‐thick aluminum filter for beam hardening. The images were reconstructed using NRecon Reconstruction software (Bruker microCT, Kontich, Belgium). After that, a refined volume of the 0.5 mm below the growth plate and 1 mm in height was chosen for further qualitative and quantitative analyses using the DATAVIEWER and CTVox software programs (Bruker microCT). Data, including the bone volume/tissue volume ratio (BV/TV), trabecular thickness (Tb.Th), trabecular number (Tb.N), and trabecular separation (Tb.Sp), were analysed by CTAn software (Bruker microCT) as previously described.^27^

### 2.12 Histological and histomorphometric analysis

The right femur samples were fixed in 4% paraformaldehyde for 48 h and then decalcified in 14% ethylenediaminetetraacetic (EDTA) at 37 degree for 10 days. After decalcification, the samples were embedded in paraffin and cut in the sagittal plane to produce approximately 5‐μm‐thick sections. Hematoxylin and eosin (H&E) and TRAcP staining were performed as described previously.^27^ Images of the stained sections were examined using uScope MXII Digital Microscope Slide Scanner (Microscopes International, Lubbock, TX, USA), and representative images were collected. Claret‐red granules in the vicinity of the resorbed bone were counted as TRAcP‐positive cells. The OC number per bone surface (N.Oc/BS) and the osteoclast surface per bone surface (Oc.S/BS) were calculated using BIOQUANT OSTEO 2011 software according to a method proposed by Sawyer et al.^33^

### 2.13 Statistical analysis

The results are presented as the means ± standard deviation (SD). The significance of difference between two groups was determined by Student's *t* test. One‐way ANOVA plus Tukey's test or Kruskal‐Wallis analysis (non‐parametric ANOVA) plus Dunn's multiple comparison (when the data failed the assumptions of the one‐way ANOVA) were used to test differences between more than two groups. A two‐way ANOVA was conducted to examine the effects of time and different treatment groups. *P* < 0.05 was considered statistically significant.

## 3 RESULTS

### 3.1 MA inhibits RANKL‐induced osteoclast formation in a dose‐dependent manner

**Figure 1:**
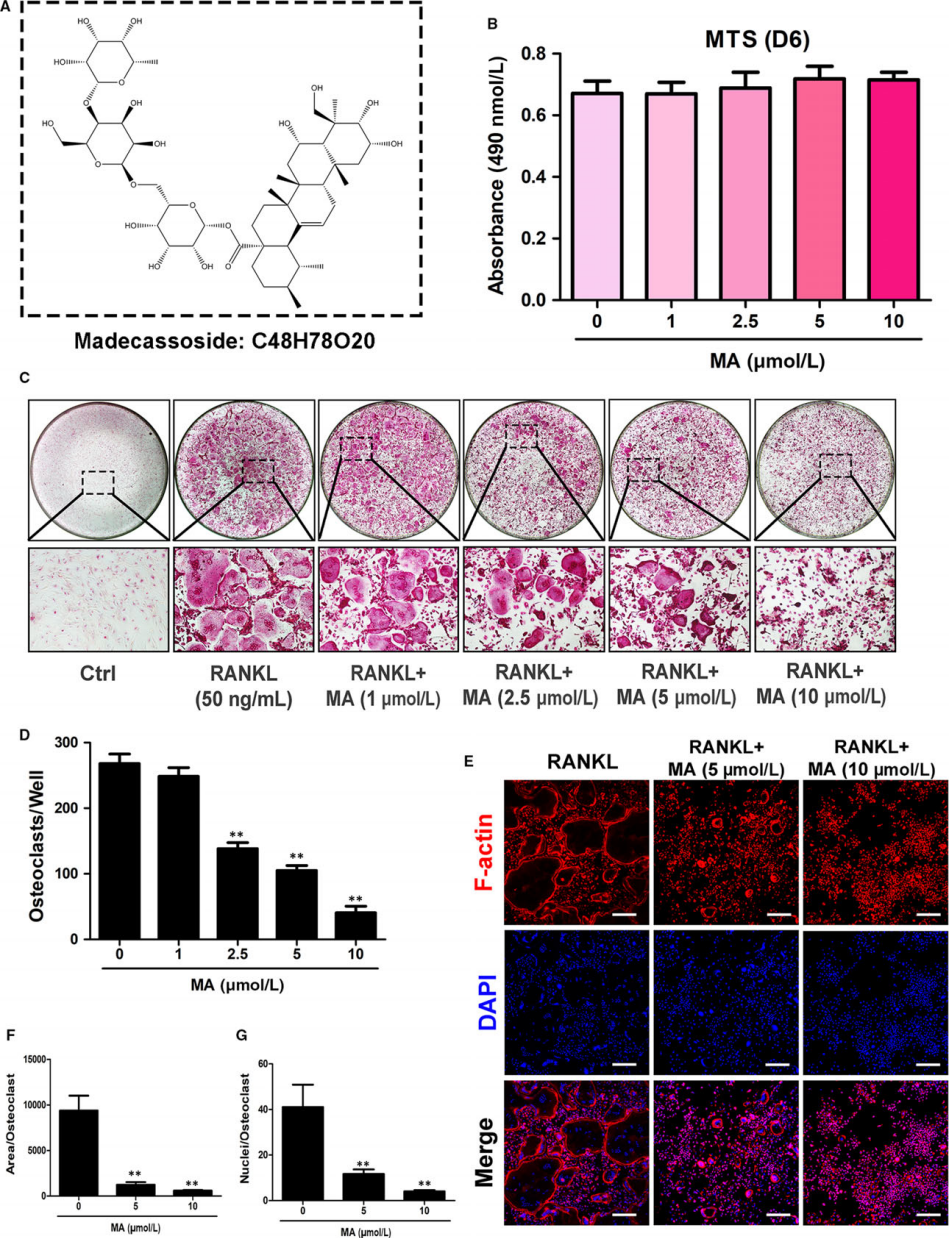
Madecassoside (MA) suppresses RANKL‐induced osteoclast differentiation in a dose‐dependent manner. (A) Chemical structure of MA. (B) The effects of the indicated concentrations of MA on BMMs were measured by an MTS assay. (C) Representative images of OCs after treatment with MA at increasing concentrations (magnification = 100×). (D) The number of TRAcP^+^ multinucleated cells (>3 nuclei) per well (96‐well plate) was quantitatively analysed. (E) Representative confocal images of OCs stained for F‐actin and nuclei; the images include untreated OCs and OCs treated with 5 μmol L^−1^ and 10 μmol L^−1^ Madecassoside. Scale bar = 200 μm. (F‐G) Quantification of the OCs per area and the mean number of nuclei in each cell. Data are expressed as the means ± SD; *P < 0.05, **P < 0.01, and ***P < 0.001 compared to control group

**Figure 2:**
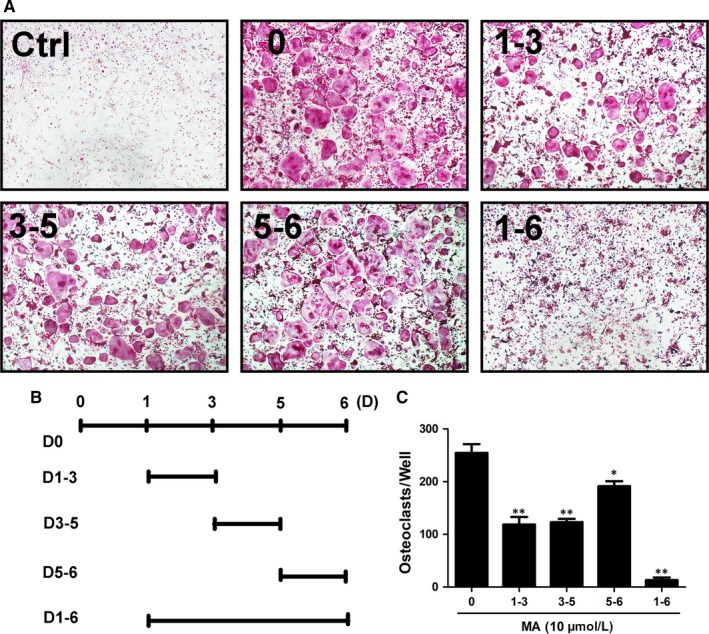
Madecassoside (MA) suppresses RANKL‐induced osteoclastogenesis in the early stages. (A‐B) Representative images of TRAcP^+^ cells treated with 10 μmol L^−1^ MA on the indicated days (magnification = 4×). (C) TRAcP‐stained cells (>3 nuclei) treated with MA at different time periods were quantitatively analyzed for osteoclast formation. Data are presented as the means ± SD; *P < 0.05, **P < 0.01, and ***P < 0.001 relative to RANKL‐induced controls

Numerous traditional Chinese medicines were added to the osteoclastogenesis assay as candidates to screen their ability to inhibit the RANKL‐induced formation of OCs from BMMs at a concentration of 10 μmol L^−1^ (Table 2). Moreover, an MTS assay was performed to measure the cell cytotoxicity of these natural compounds, which might be confused with their effect on the suppression of OC formation. As can be seen from Table 2, MA was found to significantly inhibit osteoclast formation at a concentration of 10 μmol L^−1^, although most of the other compounds had no effect on suppressing osteoclastogenesis. Furthermore, MA did not decrease the number of BMMs compared to that of the control group, an extremely encouraging result that confirmed that the inhibitory effect of MA on the generation of OCs from BMMs was not caused by cytotoxicity (Figure 1A and B). Next, to examine whether the suppressive effect of MA is dose dependent, BMMs were treated with increasing concentrations of MA (1 to 10 μmol L^−1^) in the presence of 50 ng/mL RANKL for 5 days and were stained with TRAcP buffer to visualize the formation of OCs. What is clearly evident from Figure 1C and D is that the number of TRAcP‐positive cells significantly decreased in a dose‐dependent manner in each well at concentrations of MA higher than 2.5 μmol L^−1^. OC nuclear fusion is also an important step in the formation of mature OCs. To further test the effect of MA on OC fusion, the cytoskeleton and cell nucleus were stained to observe the number of nuclei per osteoclast. Figure 1E‐G shows that both the average area of each OC and the number of nuclei per OC were notably reduced in the presence of MA at 5 and 10 μmol L^−1^. These results were consistent with the results obtained from TRAcP staining. Based on these results, further experiments were conducted to identify when the inhibitory effect of MA started to work. As shown in Figure 2A‐C, BMMs were treated with MA for several different time periods (1‐3, 3‐5, 5‐6, and 1‐6 days). TRAcP‐positive cells were significantly decreased when MA was present on days 1‐3 or 3‐5, whereas the effects were weakened when MA was present on days 5‐6, indicating that MA plays an inhibitory role in the early and middle stages of OC differentiation. In conclusion, our findings illustrated that MA abrogated RANKL‐induced osteoclast formation throughout the whole process, especially at stages that exhibited dose dependence, but did not cause cell cytotoxicity.

**Table 2:** The inhibitory effect of natural compounds on RANKL‐induced osteoclastogenesis

Compounds name	Origins	Inhibitory effect on RANKL‐induced osteoclastogenesis
Madecassoside	Natural	IC50 ≈ 5 μmol L^−1^
Shikonin	Natural	Toxic
Gentiopicroside	Natural	IC50 > 10 μmol L^−1^ or no effect
Stachydrine hydrochloride	Natural	IC50 > 10 μmol L^−1^ or no effect
Calceolarioside B	Natural	IC50 > 10 μmol L^−1^ or no effect
Dicoumarol	Natural	IC50 > 10 μmol L^−1^ or no effect
Alantolactone	Natural	IC50 > 10 μmol L^−1^ or no effect

IC50, half maximal inhibitory concentration.

### 3.2 MA attenuates osteoclast resorptive activity

**Figure 3:**
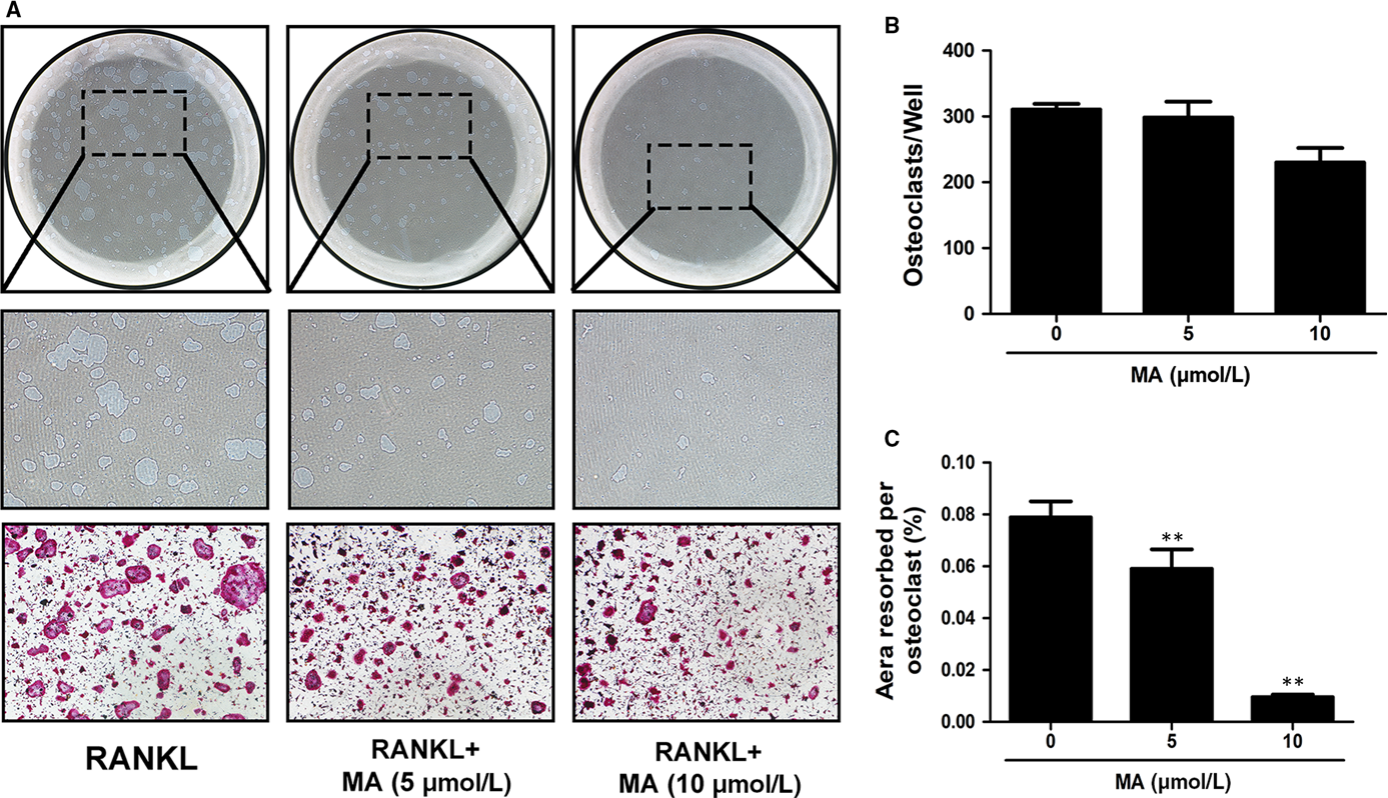
Madecassoside (MA) suppresses osteoclastic bone resorption activity. (A) Representative images of eroded areas and TRAcP‐stained cells on hydroxyapatite‐coated plates in the presence or absence of 10 μmol L^−1^ MA (magnification = 4×). (B) Quantitative analysis of the TRAcP^+^ cells in each well (96‐well plate). (C) The resorbed proportion per osteoclast after treatment with the indicated concentrations of MA was quantified. Data are presented as the means ± SD; *P < 0.05, **P < 0.01, and ***P < 0.001 relative to RANKL‐induced controls

Beyond the formation of OCs, we further investigated whether MA could attenuate the cellular resorptive function of OCs using a hydroxyapatite resorption assay. After mature OCs were transferred and treated with MA at a dose of 5 or 10 μmol L^−1^ for 2 days, the percentage of resorption area and the number of OCs per well were measured. As depicted in Figure 3, although smaller‐sized OCs were observed in the MA treatment groups, the number of OCs in each well exhibited no significant differences among all the groups, which is consistent with the above conclusion that MA inhibits osteoclastogenesis mainly in the early and middle stages. However, the resorption area decreased with increasing drug concentrations, especially at 10 μmol L^−1^, which indicates that MA can also attenuate the resorption activity of OCs apart from osteoclast formation.

### 3.3 MA suppresses the expression of genes related to RANKL‐induced OC formation and function

**Figure 4:**
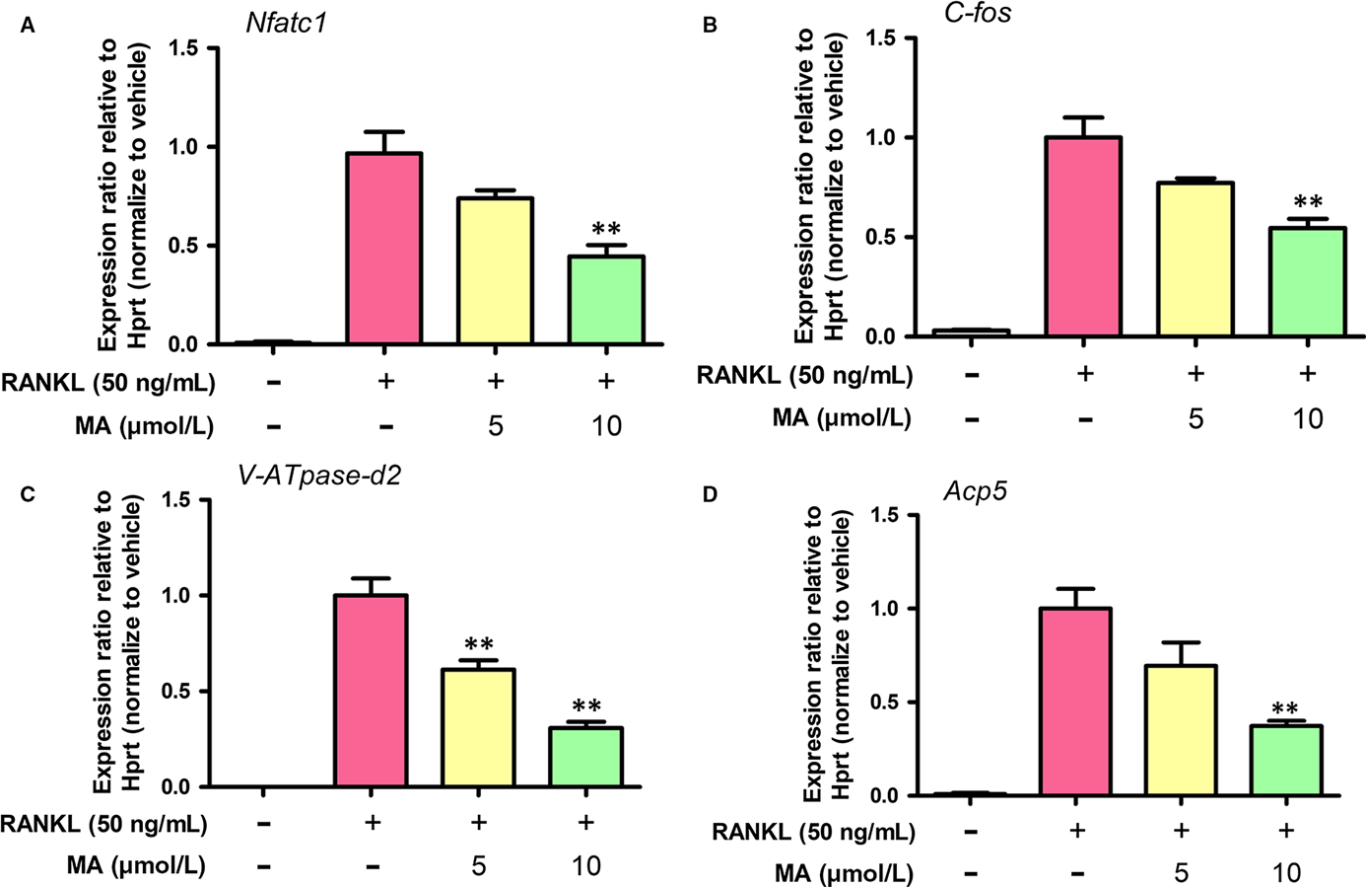
Madecassoside (MA) blocks osteoclast‐specific gene expression. (A) NFATc1, (B) c‐Fos, (C) V‐ATPase‐d2 (ATP6V0D2), and (D) Acp5 (TRAcP). Gene expression levels were standardized to Hprt expression. Data are presented as the means ± SD; *P < 0.05, **P < 0.01, and ***P < 0.001 relative to RANKL‐induced controls

Next, a qRT‐PCR assay of various key genes (e.g, Acp5 (TRAcP), ATP6V0D2, NFATc1, and c‐Fos) that play a significant role in RANKL‐induced osteoclast formation and function 18 was used to provide deeper insight into the inhibitory effect that MA exerts on osteoclastogenesis. Figure 4 shows that the expression levels of these osteoclast‐related genes were remarkably increased after stimulation with RANKL (50 ng/mL) for 5 days. However, both osteoclast formation‐regulating genes (NFATc1 and c‐Fos) and osteoclast function‐related genes (Acp5 and CTSK) were significantly downregulated by MA at the concentration of 10 μmol L^−1^ in the presence of RANKL. These results confirm the inhibitory effects of MA on RANKL‐induced OC formation and function, as described above.

### 3.4 MA represses NFATc1 activity and downstream protein expression

**Figure 5:**
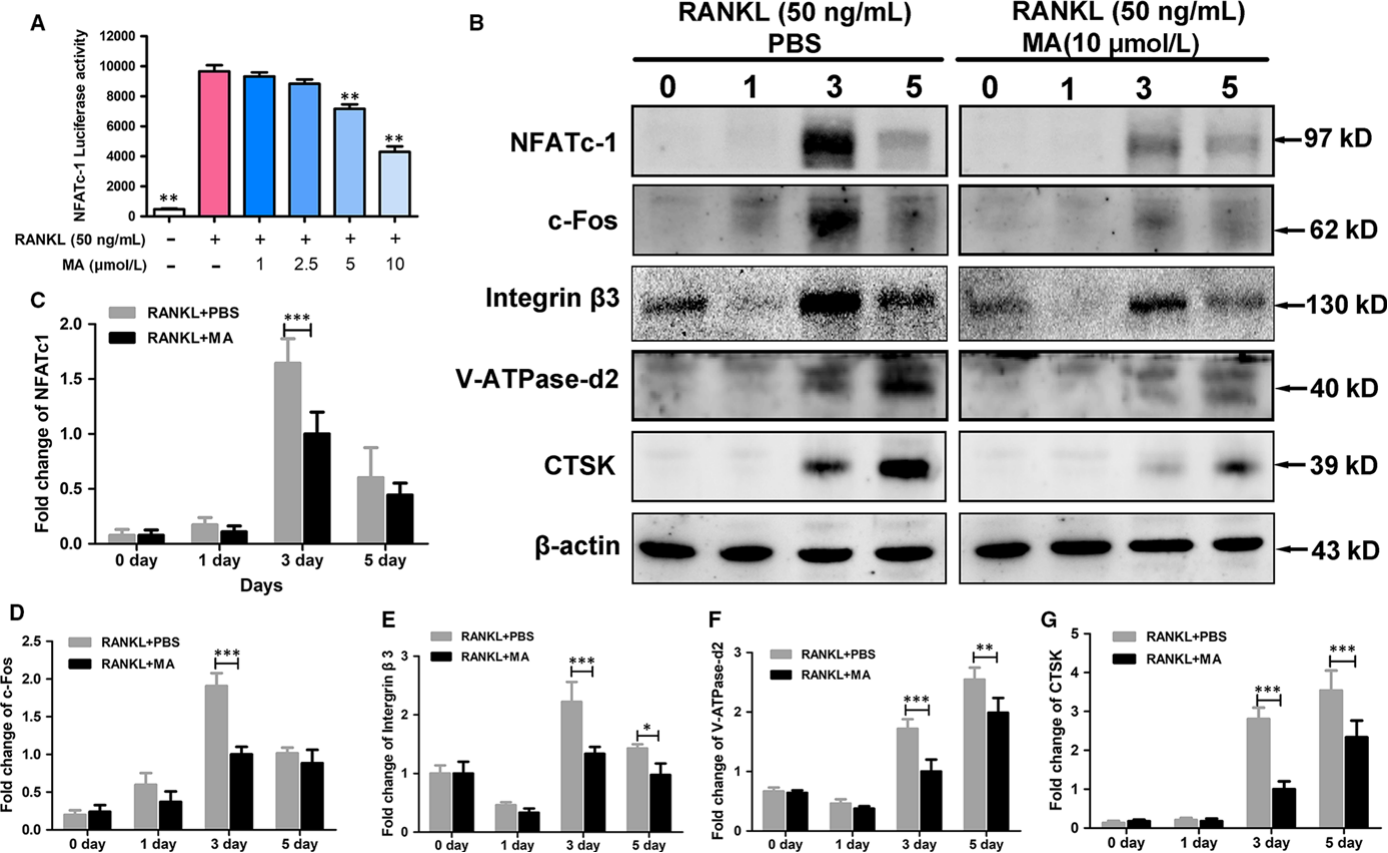
Madecassoside (MA) represses NFATc1 activity and downstream protein expression. (A) The bar graph depicts the NFATc1 luciferase activity of RAW264.7 cells stably transfected with an NFATc1 luciferase reporter construct. Cells were treated with varying densities of MA and stimulated by GST‐rRANKL (50 ng/mL) for 24 h. (B)The protein expression of NFATc1, c‐Fos, V‐ATPase‐d2, CTSK, integrin β3 at day 0, day 1, day 3, and day 5 after stimulation by GST‐rRANKL (50 ng/mL) with or without MA (10 μmol L^−1^). (C‐G) The statistical significance of differences in protein expression between the MA‐treated group and control group was analysed. The expression of all the proteins mentioned above was determined relative to β‐actin expression. The data represent the means ± SD. Significant differences between the treatment and control groups are indicated as *P < 0.05, **P < 0.01, and ***P < 0.001

To provide a complete, detailed picture of the role of MA in regulating osteoclast formation and function, a luciferase reporter assay and western blotting were chosen to detect the activity of NFATc1 and downstream protein expression. As presented in Figure 5A, the activity of NFATc1 was significantly downregulated by MA at concentrations of 5 and 10 μmol L^−1^. In addition, MA significantly inhibited the expression of the NFATc1 protein, which was remarkably upregulated 3 days after RANKL treatment (Figure 5B and C). The expression of c‐Fos, a component of AP‐1 that acts as a regulator of NFATc1, was also restricted by MA at the same time points (Figure 5B and D). Furthermore, the expression levels of downstream proteins related to osteoclast bone resorption activity, such as V‐ATPase‐d2, integrin β3, and CTSK, were also decreased, mainly 3‐5 days after MA treatment (Figure 5B and E‐G). Taken together, these findings indicated that MA produced significant inhibition of the gene and protein expression as well as activity of transcription regulators (NFATc1 and c‐Fos) that are indispensable in osteoclast differentiation and thus repressed the expression of downstream osteoclast function‐specific genes and proteins.

### 3.5 MA suppresses NF‐κB activation and calcium oscillation

**Figure 6:**
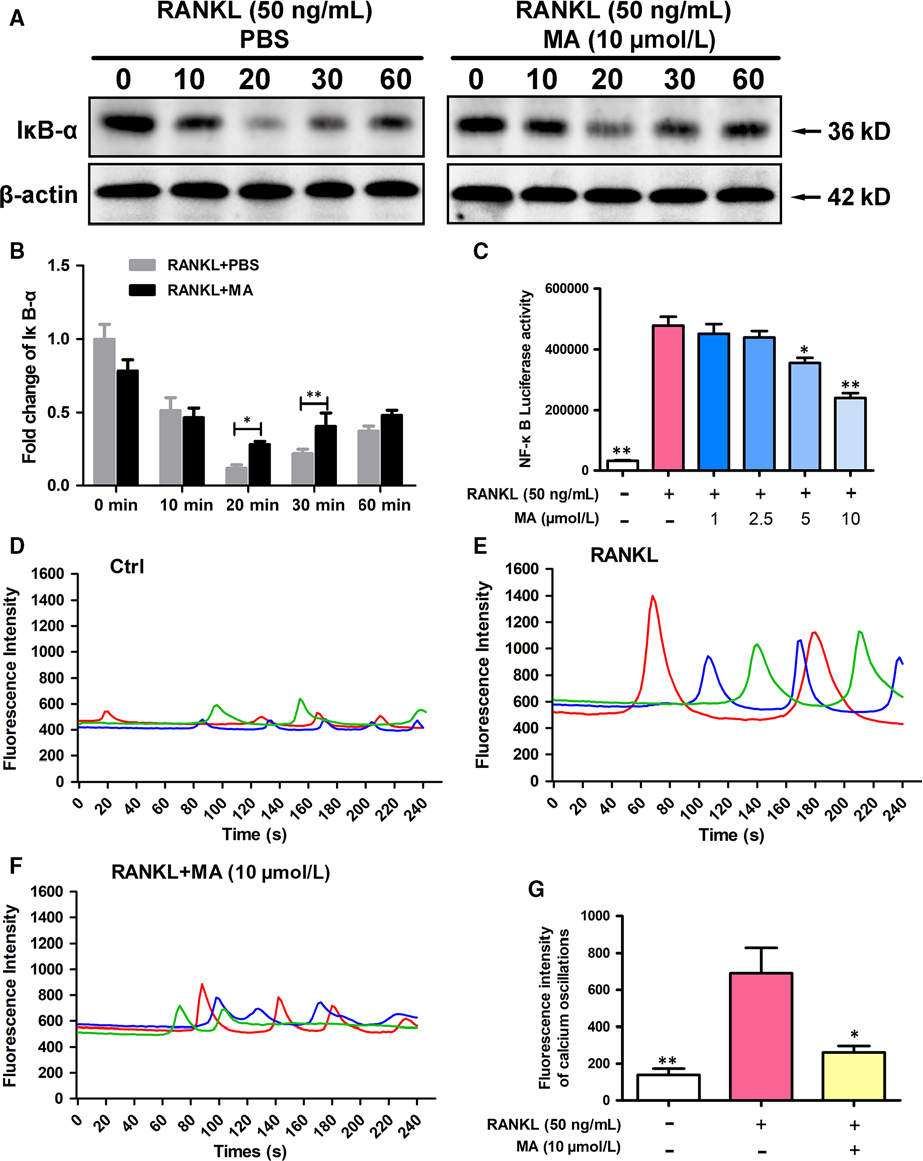
Madecassoside (MA) suppresses NF‐κB activation and calcium oscillation during osteoclastogenesis. (A) Representative images of western blots reflecting the expression level of IĸB‐α normalized to β‐actin. (B) Quantitative analysis of the fold change in IĸB‐α expression after MA (10 μmol L^−1^) treatment. (C) The bar graph depicts the NF‐κB luciferase activity of RAW264.7 cells stably transfected with an NF‐κB luciferase reporter construct. Cells were treated with varying densities of MA and stimulated by GST‐rRANKL (50 ng/mL) for 6 h. (D) Representative images of Ca^2+^ oscillation patterns without stimulation by RANKL (M‐CSF only). (E) Representative images of calcium fluctuation patterns stimulated by RANKL. (F) Representative images of calcium fluctuation patterns stimulated by MA (10 μmol L^−1^) + RANKL. (G) Quantitative analysis of the amplitude of the fluorescence intensity of calcium oscillations in each group. Lines with different colours in each image represent the results of three independent experiments. The data in the figures represent the means ± SD. Significant differences between the treatment and control groups are indicated as *P < 0.05, **P < 0.01, and ***P < 0.001

The transcription regulator NFATc1 plays a critical role in osteoclast differentiation.^34^ To gain a detailed understanding of the molecular mechanisms underlying the regulation and activation of NFATc1, the NF‐κB and Ca^2+^ signaling pathways were measured using a luciferase assay, western blotting, and calcium oscillation.^35^, ^36^ IκB is a major signaling molecule related to the activation of NF‐κB. As shown in Figure 6A and B, the degradation of IκB was inhibited 20‐30 min after treatment with MA compared to the RANKL‐only group. Furthermore, the results shown in Figure 6C suggested that the activation of NF‐κB was remarkedly increased by RANKL stimulation and suppressed by MA in a concentration‐dependent manner at concentrations above 5 μmol L^−1^, which further proved the ability of MA to suppress NF‐κB activation during RANKL‐induced NFATc1 activation. Ca^2+^ oscillations, which are initiated by RANKL‐stimulated Ca^2+^ signal transduction pathways, also contribute to the activation of NFATc1.^35^ Hence, the effect of MA on Ca^2+^ oscillations was further examined (Figure 6D‐G). Consistent with our expectations, the level of calcium oscillations induced by RANKL was dramatically decreased, by nearly 60%, in the presence of MA (10 μmol L^−1^). Taken together, these findings suggest that the strong inhibitory effect of MA on NFATc1 activation could be due to the prevention of both RANKL‐mediated NF‐κB activation and Ca^2+^ oscillations.

### 3.6 MA inhibits the RANKL‐induced MAPK signaling pathway

**Figure 7:**
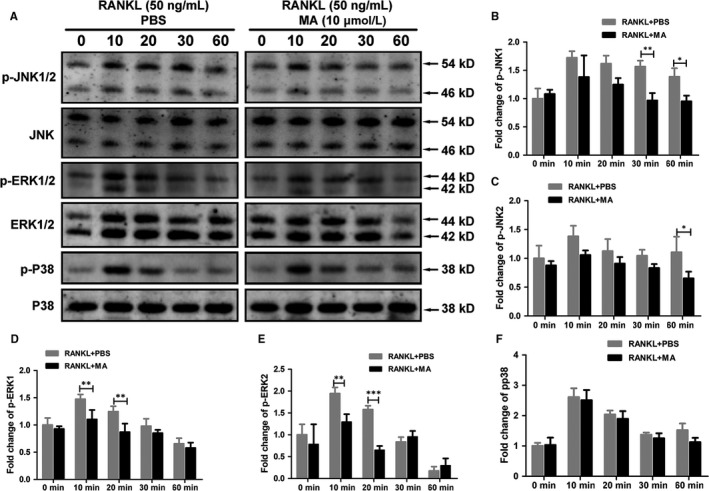
Madecassoside (MA) inhibits the RANKL‐induced MAPK signaling pathway. (A) Representative western blot images of p‐JNK1/2, JNK, p‐ERK1/2, p‐p38, p38, ERK, and β‐actin at 0, 10, 20, 30, 60 min stimulated by GST‐rRANKL (50 ng/mL) with or without MA (10 μmol L^−1^). (B‐F) The relative ratios of phosphorylated proteins to unphosphorylated proteins were quantitatively determined. The data in the figures represent the means ± SD. Significant differences between the treatment and control groups are indicated as *P < 0.05, **P < 0.01, and ***P < 0.001

c‐Fos (a member of the AP‐1 family) is an indispensable transcriptional activator of the regulation and induction of NFATc1 in the process of osteoclast differentiation.^37^ Members of the MAPK signaling pathway (p38, JNK, and ERK), which plays a crucial role in regulating AP‐1 activity,^38^ were measured at 0, 10, 20, 30, 60 min after RANKL stimulation with or without MA. As depicted in Figure 7, MA remarkably suppressed the phosphorylation of JNK at 30 and 60 min after RANKL treatment. In addition, the expression levels of p‐ERK1 and p‐ERK2 in BMMs were significantly decreased by MA after 10 and 20 min of co‐incubation with RANKL. However, no differences in p‐p38 expression were found between the groups with and without MA. These findings suggested that MA can inhibit the RANKL‐induced MAPK signaling pathway, especially the expression of p‐JNK and p‐ERK1/2, and they provide further support for the inhibitory effect of MA on c‐Fos.

### 3.7 MA prevents estrogen deficiency‐induced bone loss in vivo

**Figure 8:**
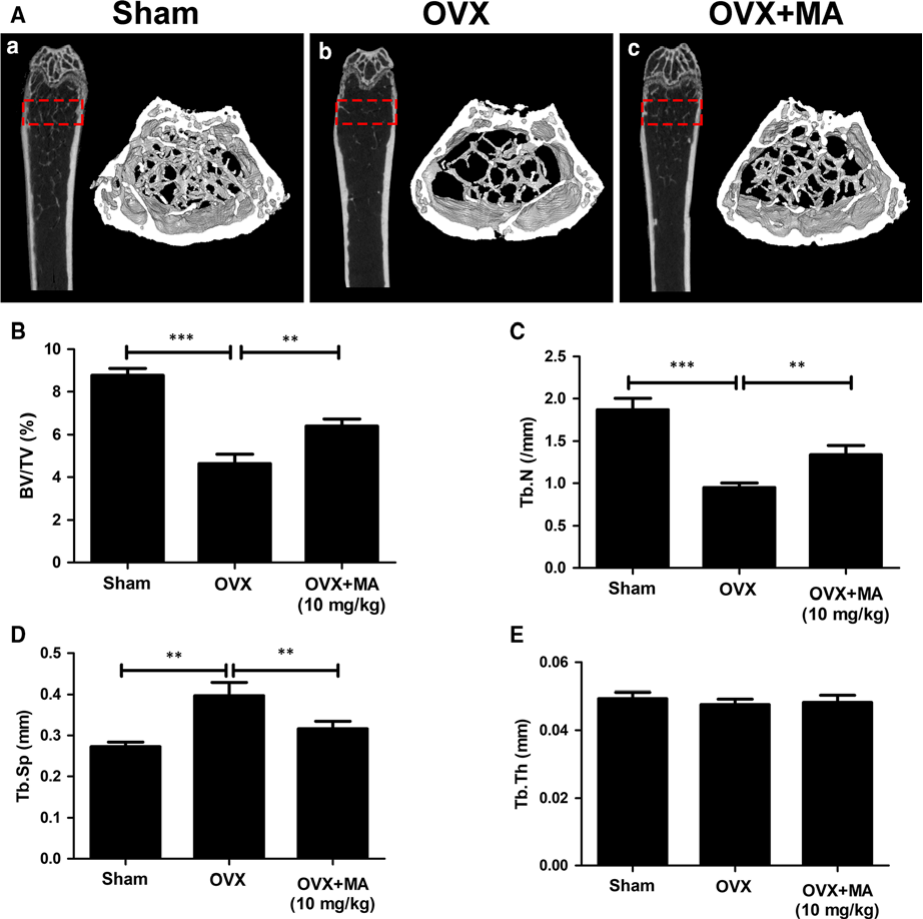
Madecassoside (MA) ameliorates OVX‐induced systematic bone loss. (A) Femur structure captured by high‐resolution μCT and post‐processed by 3D computer reconstruction. (B‐E) Quantitative measurements of bone microstructure‐related parameters, such as BV/TV, Tb.N, Tb.Th, and Tb.Sp, amongst the Sham+Vehicle, OVX+Vehicle, and OVX+MA (10 mg/kg) groups. The data in the figures represent the means ± SD. Significant differences between the treatment and control groups are indicated as *P < 0.05, **P < 0.01, and ***P < 0.001

To further evaluate the therapeutic value of MA in osteolytic diseases, we conducted animal experiments using an estrogen deficiency‐induced osteoporosis mouse model treated with either vehicle or MA every 2 days for 6 weeks. Micro‐CT and histomorphometric assessments were then separately performed for qualitative and quantitative analyses of several bone parameters that reflect the effect of MA on maintaining bone mass. The micro‐CT results demonstrated that MA plays a strong role in preventing bone loss, with increased BV/TV and Tb.N compared to the control groups (Figure 8A‐C). Furthermore, the trabecular spacing (Tb.Sp) was also significantly decreased after MA treatment (Figure 8D). In addition, no statistically significant difference in Tb.Th was found in all groups, indicating that the trabecular thickness was not susceptible to the estrogen deficiency (Figure 8E). Taken together, these results indicated that MA exerts a protective effect on preventing estrogen deficiency‐induced bone loss.

**Figure 9:**
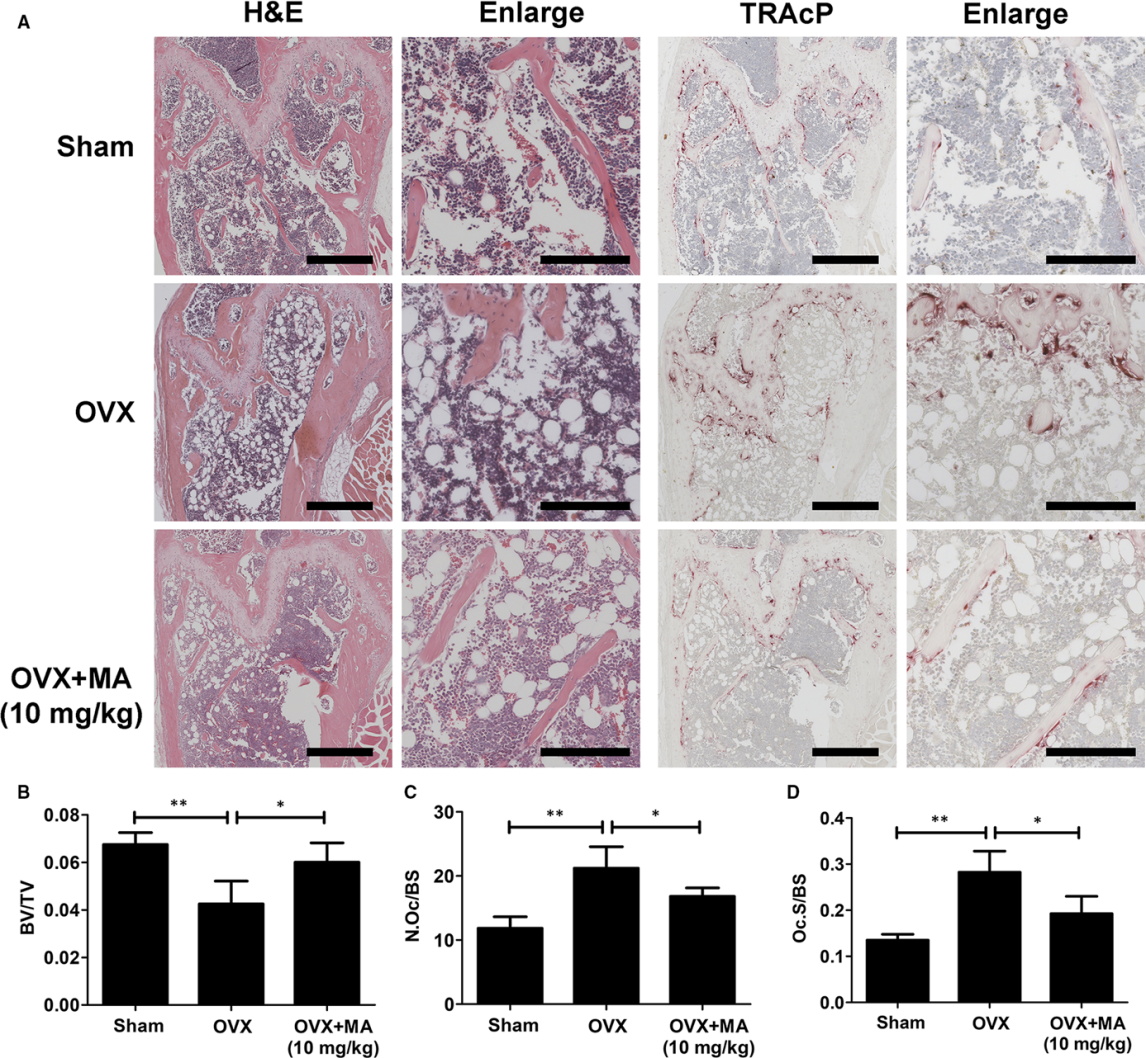
Madecassoside (MA) protects against OVX‐induced bone loss via inhibiting osteoclast activity. (A) Representative images of decalcified bone stained with H&E and TRAcP from sham mice, OVX mice, and OVX mice treated with 10 mg/kg MA. Scale bar = 500 mm; scale bar = 200 mm in the enlarge pictures. (B) Quantitative analyses of bone volume/total volume (BV/TV), osteoclast surface/bone surface (Oc.S/BS) and osteoclast number/bone surface (N.Oc/BS). *P < 0.05 and **P < 0.01

Histomorphometric assessment was further applied to increase the credibility of the results (Figure 9). The BV/TV value of the OVX+MA group was obviously higher than that of the OVX group. Other bone evaluation parameters, such as the Oc.S/BS and the N.Oc/BS decreased dramatically after treatment with MA (*P* < 0.05, compared to the OVX+PBS group). Overall, our findings suggested that MA has therapeutic value in protecting against systematic bone loss in both animal experiments and histomorphometric assessments.

## 4 DISCUSSION

**Figure 10:**
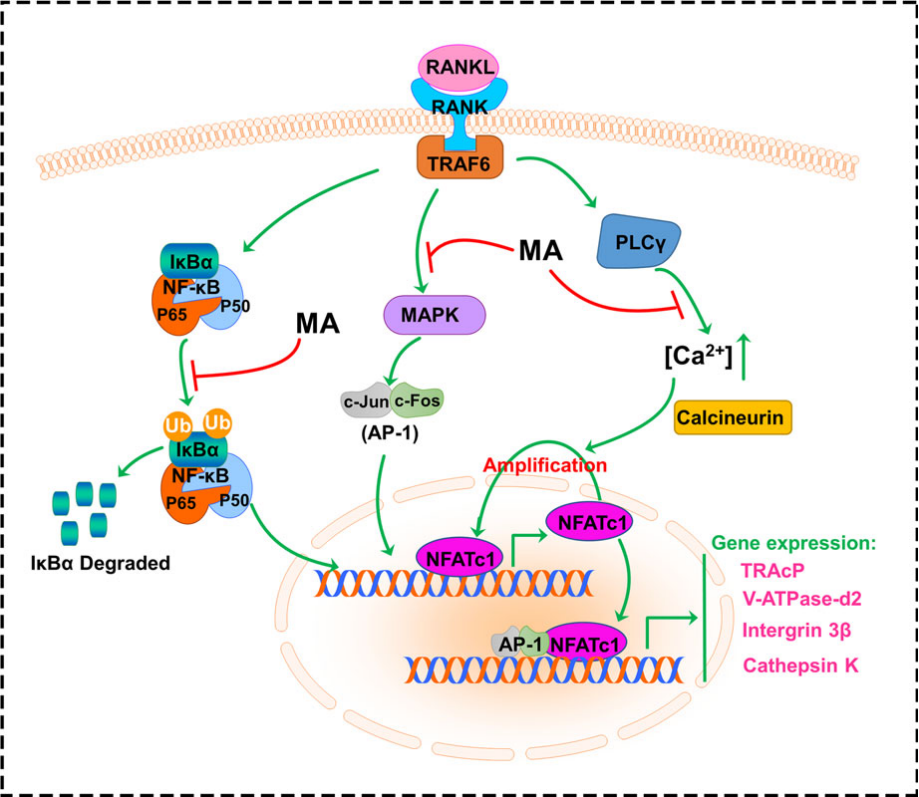
A schematic diagram helps in understanding the role of Madecassoside in suppressing RANKL‐induced osteoclastogenesis

Based on the fact that current treatments for skeletal lytic diseases are accompanied by side effects, including osteonecrosis, and hormonal disorders,^7^ which limit their clinical application, new therapeutic strategies to prevent osteolytic disease are urgently needed. Renewed interests in natural compounds have increased in recent years because of their broad‐spectrum of biological activity and fewer side effects.^9^, ^39^, ^40^ Here, we screened numerous natural compounds that have been reported to possess one or more bioactivities, such as antioxidative, anti‐inflammatory, and antibacterial activities, for osteoporosis treatment at clinically acceptable concentrations.^23^, ^41^, ^42^ We found that MA, which is characterized as a triterpenoid derivative extracted from *Centella asiatica*, showed a remarkable effect on inhibiting RANKL‐induced osteoclastogenesis. MA has been widely used in Chinese traditional medicine and it was reported to exert numerous bioactivities, such as attenuating the inflammatory response in collagen‐induced arthritis in DBA/1 mice 23 and reducing ischemia‐reperfusion injury in regional ischemia‐induced heart infarction.^43^ Furthermore, a clinical trial showed that MA had a long‐term effect on photooxidative ageing of human skin by modulating inflammatory mediators, which provided practical proof of its safety for clinical use.^42^ In this study, as shown in Figure 10, we demonstrated that MA not only inhibited the formation of OCs but also decreased the bone resorption activity of OCs by affecting the activity of transcription factors in the NF‐κB and MAPK signaling pathways. Furthermore, we showed a protective effect of MA against bone loss in vivo by using an estrogen deficiency‐induced osteoporosis mouse model. The findings demonstrated that MA has a great potential value in the treatment of osteoporosis.

OCs are multinucleated cells derived from the fusion of precursor cells; this fusion process is impaired in the absence of V‐ATPase‐d2.^19^ Bone absorption is an important function of OCs in the body. Once OCs attach to the membrane of the bone surface with the engagement of the adhesion receptor integrin β3,^44^ a sealing zone forms to promote firmer attachment.^45^ Then, the OCs secrete numerous enzymes, such as CTSK and TRAcP. CTSK plays a major role in degrading the bone matrix, while TRAcP mainly enhances the activity of CTSK.^46^, ^47^ In our study, the gene and protein expression levels of CTSK, Acp5 (TRAcP), V‐ATPase‐d2, and integrin β3 were found to be significantly inhibited after treatment with MA, which contributed to the downregulation of RANKL‐induced osteoclastic bone resorption.

The bone resorption‐related genes and proteins mentioned above are regulated by NFATc1. NFATc1 is an indispensable transcription factor that acts in the formation of OCs from BMMs.^17^, ^48^ Our results displayed a significant reduction of both the gene and protein expression and the activity of NFATc1, which confirmed that this crucial factor was remarkably suppressed by MA treatment. NF‐κB acts as an initiator of NFATc1 induction during RANKL‐induced osteoclastogenesis. It was reported that after treatment with dehydroxymethylepoxyquinomicin (DHMEQ) (an NF‐κB inhibitor), the activity of NFATc1 was significantly suppressed.^49^ Another experiment demonstrated that p50 and p65 (two components of NF‐κB) activated the NFATc1 promoter 1 hour after RANKL interacted with RANK, illustrating the close relationship between NF‐κB and NFATc1.^50^, ^51^ In the cytoplasm of non‐stimulated cells, NF‐κB exists in a complex with IκB. IκB is then degraded by inhibitor of κB kinase (IKK) after RANKL stimulation, whereas NF‐κB enters the nucleus and stimulates the transcription of key genes.^52^ Therefore, IκB can be regarded as a signaling molecule that reflects NF‐κB activation. As is shown in our study, both the activation of NF‐κB and the degradation of IκB were repressed by MA. Previous studies found that the activation and auto‐amplification of NFATc1 were mediated by Ca^2+^ oscillations induced by RANKL, which demonstrated that NFATc1 was also regulated by the Ca/calcineurin pathway.^35^ Combined with the Ca^2+^ oscillation results, the strong effect of MA on inhibiting NFATc1 and thus preventing the formation of OCs suggests a dual role in blocking the NF‐κB and Ca/calcineurin pathways.

c‐Fos (an AP‐1 component) is an indispensable factor that triggers a transcriptional regulatory cascade by producing and cooperating with NFATc1, thereby activating a number of target genes involved in osteoclast differentiation and function.^17^ This finding was confirmed by a study that found the expression of RANKL‐induced NFATc1 was abrogated in c‐Fos knockout mice.^37^ In addition, another study demonstrated that mice developed osteopetrosis because of c‐Fos deficiency, which also showed the importance of c‐Fos in RANKL‐induced osteoclastogenesis.^53^ In our study, the gene and protein expression of c‐Fos was significantly inhibited by MA. MAPK, which consists of JNK, ERK, and p38, is part of the RANKL‐induced signaling pathway that regulates the expression of AP‐1.^54^ It has been reported that ERK contributes to the protection of OCs from apoptosis and the stimulation of osteoclast differentiation,^55^ which explains the increase in cell number after RANKL treatment. The blockade of JNK also leads to the failure of osteoclast formation. Although p38 is not involved in osteoclast function, this protein does participate in osteoclast differentiation.^56^ Our western blot results showed that MA first inhibited the phosphorylation of ERK1/2 (at 10 and 20 minutes) and then suppressed the phosphorylation of JNK1/2 (at 30 and 60 minutes) after stimulation by RANKL. Therefore, we propose that MA can regulate the activation of AP‐1 by interfering with MAPK signaling and inhibiting the subsequent induction of NFATc1.

In conclusion, our study indicated that MA can inhibit RANKL‐induced osteoclastogenesis via NFATc1 by targeting the NF‐κB, Ca/calcineurin and MAPK signaling pathways. Furthermore, with the satisfactory therapeutic effect of MA on estrogen deficiency‐induced osteoporosis, which was first described and confirmed in this study, MA, a natural compound extracted from a traditional Chinese herb, may be a potential therapeutic candidate for preventing and treating osteoporosis.

## CONFLICT OF INTEREST

The authors declare no conflict of interest.
